# Neck dissection: an operation in evolution

**DOI:** 10.1186/1477-7819-3-22

**Published:** 2005-04-18

**Authors:** Ashok R Shaha

**Affiliations:** 1 Head and Neck Service, MSKCC 1275 York Avenue New York, NY 10021, USA

## 

The most important prognostic factor in the management of head and neck cancer is the presence of cervical nodal metastasis. Once the tumor involves neck nodes, survival drops by almost 50%. Management of cervical metastasis has gone through an evolution since the beginning of the last century. The classic radical neck dissection, where all the neck nodes are removed along with 3 important structures – sternocleidomastoid muscle, internal jugular vein and accessory nerve – was popularized in the landmark article by George Crile. It subsequently became the standard of care in the management of neck nodes for almost 75 years.

The major complication from radical neck dissection was very apparent to most clinicians; shoulder dysfunction, which led to modifications in neck dissection techniques. Oswaldo Suarez gets the credit for popularizing functional neck dissection wherein the accessory nerve is carefully preserved to the extent tumor involvement allows. He also popularized the facial envelope and oncologic safety of modified neck dissection, which gained acceptance with the teachings of Itore Bocca, Javier Gavilan, and Richard Jesse. In the 1980's there was a tremendous switch from radical neck dissection to modified neck dissection to maintain patient quality of life and preserve shoulder function. At the same time, the patterns of nodal metastasis were studied in detail. Publications from Lindberg and Shah described the location of metastatic disease in the neck depending upon the primary site, and justified modifications in neck dissection [1]. By the mid 1980's there was confusion and disagreement about the nomenclature of various modifications, and every institution had their own modification. In an effort to standardize the nomenclature, the American Academy of Otolaryngology-Head and Neck Surgery developed a systematic approach to neck dissection, dividing it into comprehensive and selective neck dissections [2,3].

In the recent modification of the standardization of neck dissection, the Committee on Head and Neck Oncology divided Levels I, II and V into A&B groups [3]. This division appears to be anatomically sound in relation to metastatic disease to the neck. For example, Level IA is rarely involved in metastatic disease. At the same time, Level IIB nodes are rarely involved in metastatic tumor unless there is a bulky metastatic disease at Level II. One may consider super-selective neck dissections. There appears to be a trend in deleting specific names for the modification of neck dissection and use selective neck dissections with the structures and group of lymph nodes removed as specific types.

The comprehensive neck dissection removes all lymph nodes in the neck and its modification includes preservation of the sternocleidomastoid muscle, accessory nerve or jugular vein. The selective neck dissection addresses a select group of lymph nodes based on the location of the highest incidence of metastatic disease, thus supraomohyoid neck dissection became very popular as a staging procedure for cancer of the oral cavity [4]. Interest developed in understanding the prognostic factors of metastatic disease in the neck, such as tumor size, location, and extranodal spread.

Postoperative radiation therapy in patients with cervical neck node metastasis became the standard practice in the early 1990's. The high incidence of metastatic disease to neck nodes from cancer of the oral tongue was noted at the same time. Supraomohyoid neck dissection became standard practice in patients with cancer of the oral cavity and N0 neck.

Clinical evaluation was supplemented with imaging studies, such as computerized tomography (CT) and magnetic resonance imaging (MRI) scanning. Van den Brekel from Amsterdam popularized the role of ultrasound and ultrasound-guided needle biopsy [5]. Even though the researchers from Amsterdam reported excellent correlation, ultrasound did not become very popular in day-to-day clinical surgical practice. It does play an important role in the initial evaluation of cervical metastasis and in patient follow-up, particularly in those who have received chemo-radiation therapy. Ultrasound is an easy outpatient test which can be performed at frequent intervals during follow-up. In the mid 1990's an interest developed in using PET scanning to diagnose neck metastasis. Even though PET scanning can be a useful tool, particularly FDG uptake evaluation, it has not helped to make clinically definitive decisions as to the presence or absence of metastatic disease. Small volume disease, especially in the N0 neck, is difficult to image with a PET scan. PET scanning appears to be an important investigative tool in patients who are being followed after nonsurgical treatment, such as chemo-radiation therapy. Further studies are necessary, however, to standardize the SUV (Standard Uptake Value) in the PET scan.

Postoperative radiation therapy was routinely recommended in patients with large nodal metastasis and extranodal spread. Extranodal spread was considered to indicate grave prognosis in patients with cervical metastasis. These patients had a high incidence of local recurrence and distant metastasis. With this in mind, the EORTC and RTOG conducted randomized prospective trials of the use of postoperative chemo-radiation therapy in patients with cervical nodal metastasis. It is interesting that the two groups reported their results in the same issue of the New England Journal of Medicine.

Cooper, *et al*, [6] from the Radiation Therapy Oncology Group reported results from a randomized prospective trial in the New England Journal of Medicine. There were 231 patients randomly assigned to receive postoperative radiation therapy alone, and 228 patients to receive identical treatment plus concurrent chemotherapy with cisplatinum 100 mg per m^2 ^on days 1, 22 and 43. They reported the estimated 2 year rate of local and regional control as 82% in the combined therapy group, as compared with 72% in the radiotherapy alone group. Disease-free survival was significantly longer in the combined therapy group than in the radiotherapy group; however, interestingly the overall survival was not altered by the addition of chemotherapy. The incidence of acute adverse effects of Grade III or greater was reported in 34% of the radiotherapy group and 77% in the combined therapy group. Four patients who received combined therapy died as a direct result of treatment. The authors concluded that among high risk patients in the postoperative setting, concurrent chemo-radiation therapy significantly improves the rates of local and regional control and disease-free survival. However, the combined treatment is associated with a substantial increase in adverse effects.

In the same issue of the New England Journal of Medicine, Bernier *et al*, [7] reported on the European Organization for Research and Treatment Cancer Trial 22931. They randomly assigned 167 patients in each group to receive postoperative radiation therapy or radiation and chemotherapy. They also used 100 mg cisplatinum per m^2 ^on days 1, 22 and 43 of the radiotherapy regimen. They reported a 5 year progression-free survival of 47% compared to 36% with radiation therapy alone. They reported an overall survival rate of 53% in patients who received chemo-radiation therapy compared to 40% with radiation therapy alone. The estimated 5-year cumulative incidence of local or regional relapses was 31% after radiotherapy and 18% after combined therapy. This group also reported severe adverse effects from combined therapy (41%) and radiotherapy alone (21%).

Even though these two studies are prospective randomized trials, further confirmation needs to be obtained through continued interest in such trials including reduction of adverse effects. The most important complication of combined chemo-radiation therapy that has been noted in organ preservation protocols is severe mucositis and pharyngeal stricture. Pharyngeal stricture is a disastrous complication from this treatment and has a major impact on the quality of life of the patients.

It appears that in patients with poor prognostic factors with cervical metastasis there is an increasing interest in treatment with chemo-radiation therapy, rather than radiation alone. Obviously one needs to keep in mind the complications related to chemo-radiation therapy. Such complications are well recognized in patients who undergo an organ preservation protocol for laryngopharyngeal tumors or for oropharyngeal cancers. There is a high incidence of neutropenia, Grade 4 mucositis, and pharyngeal stricture. Development of pharyngeal stricture continues to be a difficult problem to manage in clinical practice and leads to discussion regarding the quality of life. Long term dependency on gastrostomy is extremely frustrating to patients who may have been cured of their neck disease. Since organ preservation and chemo-radiation therapy has become the most prevalent treatment for patients with oropharyngeal and laryngopharyngeal cancers, it has generated controversy about the management of patients with nodal metastasis and their follow-up. The general consensus of opinion is that N1 disease can be easily controlled with chemo-radiation therapy. The problem comes with patients who present with N2 and N3 neck disease. Even though approximately 50% of the patients can be cured with chemo-radiation therapy, the remaining patients may persist with microscopic nodal metastasis. Even though there is no unanimous consensus today about how to manage N2–N3 neck after chemo-radiation therapy, there appears to be a general trend to consider close follow-up of these patients with clinical exam, CT, MRI and PET scan. If there is a residual thickening or presence of nodal disease, neck dissection is routinely recommended. Approximately 40–50% of patients may have viable tumor, which also depends upon the location of the primary tumor.

The patients who recur in their neck nodes after previous surgery and radiation therapy are clearly a major challenge to the head and neck surgeon. Every attempt is made to resect the tumor if that is possible, along with (in select patients) additional local radiation therapy or brachytherapy. If brachy catheters are to be used, the carotid artery needs additional protection, preferably with myocutaneous flaps. Carotid resection is considered in very select circumstances where the patient has satisfactory carotid blood flow from the opposite side, and appropriate reconstruction is considered with carotid replacement by either a gortex graft or saphenous vein. There is a high incidence of neurologic complications when the carotid artery is resected. One needs to keep in mind that the carotid artery may not be the only limiting factor in such patients with metastatic disease to the neck. The surrounding structures, such as the vagus nerve, sympathetic trunk, and scalene muscles are also directly involved by the tumor. An appropriate and satisfactory surgical resection should be undertaken only if possible.

With increasing interest in sentinel node biopsy in melanoma and breast cancer, some investigators have extended this technology to the squamous carcinoma of the upper aerodigestive tract, especially cancer of the oral cavity [8]. The American College of Surgeons Oncology Group has launched a prospective study of sentinel node biopsy in tumors of the oral cavity. At this stage, the sentinel node biopsy should be used only as an investigational tool.

## Competing interests

The author(s) declare that they have no competing interests.

## Authors' contributions

ARS: Conceptualized the editorial prepared the draft and edited it.
